# Does the Inclusion of Free Sugars as Opposed to Total Sugars in Nutrient Profiling Models Improve Their Performance? A Cross-sectional Analysis From the PREDISE Study

**DOI:** 10.1016/j.tjnut.2025.05.038

**Published:** 2025-05-24

**Authors:** Alicia Corriveau, Mylène Turcotte, Amélie Bergeron, Simone Lemieux, Marie-Ève Labonté

**Affiliations:** 1 Centre Nutrition, santé, et société (NUTRISS), Institute of Nutrition and Functional Foods (INAF), Université Laval, Quebec City, Québec, Canada; 2 School of Nutrition, Faculty of Agriculture and Food Sciences, Université Laval, Quebec City, Québec, Canada; 3 Institut national de santé publique du Québec, Quebec City, Québec, Canada

**Keywords:** nutrient profiling models, Health Star Rating, Nutri-Score, NRF, validation study, cross-sectional study, diet quality, Healthy Eating Food Index, HEFI-2019, PREDISE

## Abstract

**Background:**

Nutrient profiling (NP) models characterize the healthfulness of foods. Few NP models have been validated, and nutrients included in their algorithm do not always reflect the most recent scientific evidence.

**Objectives:**

This study aimed *1*) to evaluate the validity of NP models against a diet quality measure and cardiometabolic risk factors in French-Canadians and *2*) to compare the validity of each model when replacing total sugars by free sugars in their algorithm.

**Methods:**

The PRÉDicteurs Individuels, Sociaux et Environnementaux cross-sectional study was used to test original and modified versions (i.e., including total or free sugars, respectively) of 3 NP models: Health Star Rating (HSR) system, Nutri-Score, and Nutrient-Rich Food index 6.3. Data from web-based self-administered 24-h recalls completed by 1019 adults were used to calculate energy-weighted NP-derived individual scores for both versions of each model. Associations between individual scores and the Healthy Eating Food Index 2019, as well as 14 biomarkers covering anthropometry, blood pressure, blood lipids, glucose homeostasis, and inflammatory biomarkers, were assessed using multivariable linear models.

**Results:**

Higher quality of foods consumed, as assessed by all 3 original models, was associated with higher Healthy Eating Food Index 2019 (adjusted *R*^2^: 0.43–0.55), and with lower body mass index (BMI) (β: from –0.16 to +0.48 kg/m^2^; *P* ≤ 0.0001), diastolic blood pressure (β: –0.08 to +0.30 mm Hg; *P* ≤ 0.04) and triglycerides (β: –0.01 to +0.02 mmol/L; *P* ≤ 0.002). Original HSR and Nutri-Score were also associated with lower waist circumference and HOMA-IR, lower insulin (HSR only), and higher HDL cholesterol (Nutri-Score only). Replacing total sugars by free sugars in each model only slightly increased the number of associations observed with biomarkers.

**Conclusions:**

All 3 models are associated with diet quality and some biomarkers of health status in French-Canadians, although no model outranks the others. Replacing total sugars by free sugars has little to no effect on NP models’ performance, therefore not supporting this approach for now.

## Introduction

Noncommunicable diseases (NCDs) have become a major health issue across the globe. The WHO reports 17.9 million deaths caused by NCDs annually [1]. Many modifiable behaviors can help prevent NCDs, such as adopting a healthy diet [2]. Interestingly, nutrient profiling (NP) is part of many nutrition-related public health strategies that aim to help consumers make healthier food choices [3]. As defined by the WHO, NP is “the science of classifying or ranking foods according to their nutritional composition for reasons related to preventing disease and promoting health” [4]. NP models are therefore used to characterize the overall nutritional quality (i.e., healthfulness) of foods and beverages considering various nutrients and/or components of public health concern (e.g., sugars, sodium, fiber, protein, and whole grains) in the intent to support the creation of food environments that promote healthy eating [3,5,6].

However, challenges remain regarding the use of NP models in different contexts. First, NP models represent measurement tools, and it is therefore essential to validate such tools prior to their use in a given population-based or research context [7]. A systematic review previously conducted by our group has highlighted that NP model validation is in its infancy in many jurisdictions worldwide [3]. It was found that less than half (i.e., 42%) of NP models used to support government-based nutrition-related public health strategies worldwide had been through some validation process [3]. Among these, most NP models had been validated simply by comparing their results (i.e., scores or classifications) with the results generated by a reference NP model. However, no gold standard currently exists in the field of NP [8,9]. In this context, NP experts recommend validating NP models against robust reference methods that include independently constructed measures of diet quality (convergent validity) and objective measures of health status (criterion-related validity) [8,9]. These are 2 forms of validity testing using population-based data, which therefore require to transform the NP models into NP-derived dietary indices at the individual level before proceeding to the analyses against the outcomes of interest. Because a healthy diet is generally associated with better health outcomes and given that a healthy diet is mainly composed of more nutritious foods, foods characterized as such by NP models should also be associated with a better diet quality and health status. Still, only 10% of government-based NP models have been tested for criterion-related validity globally [3]. To our knowledge, such an assessment has never been conducted in the province of Québec, Canada [3].

Another challenge is related to the type of nutrients included in NP model algorithms because these nutrients do not always reflect the most recent scientific evidence about the associations between nutrients and health. For instance, close to three-quarters of NP models used or endorsed by governmental bodies consider total sugars in their algorithm [3]. However, scientific evidence increasingly shows that free sugars are more associated than total sugars with unfavorable health outcomes such as overweight, obesity, and tooth decay [10]. Free sugars include sugars added to foods and beverages by the manufacturer, cook, or consumer, in addition to sugars naturally occurring in honey, syrups, fruit juices, and fruit juice concentrates [10]. Total sugars include not only free sugars but also sugars naturally occurring in minimally processed foods such as fresh fruits and vegetables or plain milk [11]. The WHO recommends limiting the intake of free sugars to a maximum of 10% of daily energy intake [10]. Given the evolving scientific evidence on sugars, it is worth questioning whether replacing total sugars by free sugars in NP model algorithms could increase the validity of such models and, therefore, further support the relevance of their use.

With the abovementioned considerations in mind, this study first aimed to evaluate both the convergent and the criterion-related validity of various NP models in a French-Canadian sample. A second objective was to compare the validity of each NP model when replacing the total sugars component by free sugars in their algorithm. We hypothesized that consuming foods of higher nutritional quality, as determined by each tested NP model, is associated to a higher overall diet quality (convergent validity) and a more favorable health status (criterion-related validity). For instance, a more favorable health status is assessed through cardiometabolic risk factors covering anthropometric measures, blood pressure, blood lipid profile, glucose homeostasis, and inflammatory biomarkers, all expected to show lower values except for HDL cholesterol and adiponectin. We also hypothesized that using free sugars as opposed to total sugars in NP model algorithms results in increased validity, as reflected by a stronger association with diet quality as well as stronger and/or a higher number of associations with various biomarkers of cardiometabolic risk.

## Methods

### Study design and population

This study used data from the cross-sectional *PRÉDicteurs Individuels, Sociaux et Environnementaux* (PREDISE) multicenter study, which aimed to identify the individual, social, and environmental factors associated with adherence to Canadian dietary guidelines within the French-speaking population of the province of Québec. Details about the PREDISE study are described elsewhere [12,13]. Briefly, recruitment occurred between August 2015 and April 2017 through random digit calling by a telemarketing firm within 5 administrative regions of the province of Québec (*Estrie*, *Saguenay-Lac-Saint-Jean*, *Capitale-Nationale/Chaudière-Appalaches*, *Montréal*, and *Mauricie*). Recruited participants were representative of the population from these 5 regions in terms of sex, age, and demographic weight. Eligible participants had to be adults aged between 18 and 65 years, be French speakers, have access to Internet and to a computer or tablet, and possess a valid e-mail address. Pregnant and lactating females as well as individuals with intestinal malabsorption were excluded from the study. Participants completed 3 web-based 24-h dietary recalls (described further) and different online questionnaires related to their socioeconomic, demographic, and lifestyle characteristics; their medical history and their level of physical activity (International Physical Activity Questionnaire) [14] over a 21-d period. Ethnicity was assessed in the socioeconomic, demographic, and lifestyle characteristics questionnaire with the question “What is your ethnic/cultural background?”.

**FIGURE 1:**
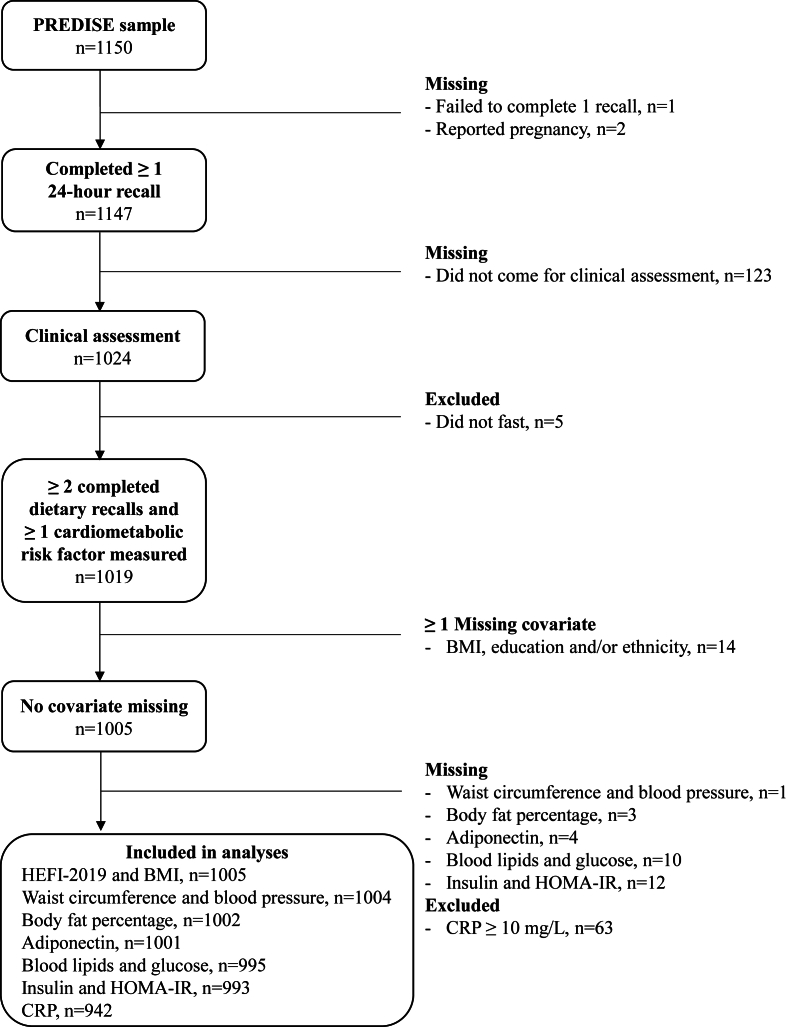
Flow chart of participants. Sample sizes are not weighted. HEFI, Healthy Eating Food Index.

Participants also went for onsite clinical assessment in their respective region where physiologic measures (e.g., weight, height, waist circumference, body fat, and blood pressure) and blood samples were taken. A total of 1147 participants were included in the final PREDISE sample. However, a maximum of 1019 participants were included in this study given that some participants did not go to their clinical assessment or did not fast prior to their appointment (Figure 1). All participants provided written informed consent. The project was conducted in accordance with the tenets of the Declaration of Helsinki and was approved by the Research Ethics Committees of Université Laval (ethics number: 2014-271), Centre Hospitalier Universitaire de Sherbrooke (ethics number: MP-31-2015-997), Montreal Clinical Research Institute (ethics number: 2015-02), and Université du Québec à Trois-Rivières (ethics number: 15-2009-07.13).

### Dietary data: 24-h web-based dietary recall

Dietary data were collected using the 24-h web-based dietary recall (R24W), a validated web-based self-administered 24-h dietary recall [[15], [16], [17], [18]]. Participants completed 3 unannounced 24-h recalls (2 weekdays and 1 weekend day) over a 21-d period. The R24W includes 2670 unique food items, of which 1209 items refer to recipes manually created using the Nutrific software from Université Laval [19]. The nutritional composition of items included in the R24W food database primarily derives from the Canadian Nutrient File (CNF) 2015 [20]. The USDA Nutrient Database for Standard Reference [21] was also used for a small percentage of items, which were not available in the CNF 2015 (5.8%).

The R24W database comprised missing values for some nutrients required in the algorithm of 1 or more of the tested NP models (described further). These missing values therefore had to be estimated to allow the calculation of a NP score for each food item using each NP model. There were missing values for energy (*n* = 1 item), saturated fats (*n* = 26), total sugars (*n* = 114), proteins (*n* = 5), fibers (*n* = 42), vitamin A (*n* = 74), vitamin C (*n* = 69), calcium (*n* = 10), iron (*n* = 13), and sodium (*n* = 5). Missing values for total sugars had already been estimated as part of previous work by Bergeron et al. [22], primarily by proportionally comparing a food item’s carbohydrate content with the carbohydrate and total sugar contents of similar foods. Missing values for the other nutrients were estimated based on the following method, which involves 3 steps. If a missing nutrient value for a given food item could not be estimated using the first step, the research team moved on to the next step and then to the third step if necessary. First, a search for a food item’s missing value was made in different databases, according to the following sequence: *1*) online version of the CNF 2015 database [20], to check whether the value was now available; *2*) the printed *Table de composition des aliments* from the province of Québec [23]; *3*) the Nutrific software [19]; and *4*) the online USDA food composition database [21]. Second, a search was made on websites of major supermarket chains found in the province of Québec [e.g., Metro, Walmart, Maxi, and IGA (*Compliments*)] to identify 1-3 commercial products corresponding to the item. The missing value was then estimated as the mean nutrient value derived from the nutrition fact table (NFt) of each of the identified commercial products. Third, when no product on supermarket websites was corresponding to the item, the NFt of 1-3 similar products (e.g., packaged chicken noodle soup instead of packaged ramen soup) was used to estimate the missing nutrient value. The estimation of missing values was conducted independently by 2 members of the research team (AC and MT). They discussed discrepancies whenever necessary, in order to reach a consensus.

### Selected NP models

Score-based NP models (i.e., generating numerical scores) were selected for this study, as these allow for a better discrimination between the overall nutritional quality of similar food products than that by category-based models (i.e., models solely generating classifications such as green, yellow, or red). This characteristic is important for a future characterization of the food supply’s nutritional quality in Québec to be conducted by the Food Quality Observatory (henceforth Observatory; https://offrealimentaire.ca/en) using NP [24]. The Observatory is a member entity of the Institute of Nutrition and Functional Foods at Université Laval, which is dedicated to monitor the food supply in the aim to generate knowledge and act collectively toward improving its quality and accessibility to the population.

NP models have been selected based on a systematic review of NP models previously conducted by Labonté et al. [3]. Briefly, all NP models originally identified in the systematic review (i.e., both included and excluded models) had to be assessed against eligibility criteria specific to the Observatory’s context (e.g., a model considered eligible had to be score based, built by a government-based or academic organization, have a publicly available algorithm, and include both nutrients to limit and to encourage) [24]. The systematic selection process of NP models, including the detailed eligibility criteria, has been described elsewhere [24]. This process led to the selection of 3 NP models to be tested in this current study, which are described further: the Health Star Rating (HSR) system [25], the Nutri-Score [26], and the Nutrient-Rich Food (NRF) index 6.3 [27].

#### HSR system

The HSR system [25] is an NP model from Australia and New Zealand, which derives from the United Kingdom’s Food Standard Agency-Nutrient Profiling System [28]. Version 6 was the version available at the time that the analyses were made. The HSR is used as a voluntary front-of-pack logo in which a calculated summary score is used to determine the number of stars assigned to a food product (i.e., between a minimum of 0.5 and a maximum of 5 stars). Application of the HSR first requires foods and beverages to be classified into 1 of the 6 categories: beverages, dairy beverages, foods, dairy foods, oils and spreads, or cheese and processed cheese. Each category has specific thresholds for nutrients to limit (energy, saturated fat, total sugars, and sodium) and nutrients or food components to encourage [protein, fiber, and percent composition of fruits, vegetables, nuts, and legumes (FVNL)], which determine the scoring points granted to each nutrient or food component [25]. Scoring points are assigned based on values per 100 g or 100 mL. A summary score is then calculated for each food product by subtracting points for nutrients to limit from points for nutrients to encourage. The scoring system ranges from −38 to 93 points, where a lower score reflects a better nutritional quality.

The estimation of FVNL points for each item in the R24W database according to the HSR guidelines was conducted following a method developed by researchers at the University of Toronto, Canada, which is based on the nature of the food item and on the ingredient list available on food packages [29]. Briefly, the FVNL content was estimated at ≤40%, >40%, >60%, >80%, or 100% and scored 0, 1, 2, 5, or 8 points, respectively. Items for which the FVNL content was considered obvious [i.e., items not containing any FVNL (0% = score of 0) such as beef, wheat flour, or plain milk, and items consisting entirely of FVNL (100% = score of 8) such as apples, plain peanuts, or raw chickpeas] were scored first. For items that likely contained a mix of FVNL and non-FVNL ingredients (e.g., breakfast cereals containing fruits and/or nuts, plant-based beverages, or soups), FVNL points were determined based upon whether the first or second ingredient in the ingredient list was considered a FVNL. If the first ingredient was a FVNL, the food product scored a 5 or a 2 depending on the small or large presence of non-FVNL ingredients; if the second ingredient (but not the first) was a FVNL, the food product scored a 1; if none of the first 2 ingredients were a FVNL, the food product scored a 0. Because ingredient lists are not available in the R24W database, products matching the item description were searched on websites of major supermarket chains found in the province of Québec (Metro, Walmart, Maxi, and IGA (*Compliments*)] to identify 1-3 commercial products corresponding to the item. These chains were chosen because they represent a wide range of supermarkets (i.e., traditional food stores and other nontraditional stores), which are popular among the studied population, and because the food products’ ingredient lists are available on their websites. FVNL content was then estimated based on the ingredient list of each product found, and if >1 product was found, the mean of the estimated FVNL content was used to assess the final FVNL score. The final calculated FVNL score was rounded down whenever necessary, as a way to be more conservative. FVNL composition of items specifically consisting of recipes (e.g., vegetable lasagna, chicken fried rice, or beef tacos) in the R24W database (*n* = 1209) was determined by using information about all ingredients entering in the composition of each recipe available in the Nutrific software [19]. More specifically, the sum of FVNL ingredients (in grams) of a given recipe was divided by the total weight (in grams) of that recipe, and then multiplied by 100 to obtain its’ percent FVNL composition.

Classification into 1 of the 6 HSR categories, determination of FVNL points, and calculation of HSR scores, for each item from the R24W database has been completed independently by 2 members of the research team (AC and MT). At each of these steps, results were compared, and discrepancies were discussed whenever necessary, to reach a consensus.

#### Nutri-Score

The Nutri-Score [30] is another NP model derived from the Food Standard Agency-Nutrient Profiling System [31]. Version 2019 was the version available at the time that the analyses were made. It was developed in France by the Haut Conseil de la santé publique in the context of their Programme National Nutrition Santé (PNNS) [32]. It is used as a voluntary front-of-pack logo in many European countries, in the form of a 5-level color scale (from green to dark orange) accompanied with letters A to E. The classification of a food product into 1 of the 5 levels is determined based on a calculated summary score. Application of the Nutri-Score first requires to classify foods and beverages into 1 of the 4 categories: beverage, food, cheese, or added fats. Similar to the HSR, each category has specific thresholds for nutrients to limit (energy, saturated fat, total sugars, and sodium) and nutrients or food components to encourage (protein, fiber, and FVNL composition), which determine the scoring points granted to each nutrient or food component [31]. Scoring points are assigned based on values per 100 g or 100 mL. A summary score is then calculated for each food product by subtracting points for nutrients to limit from points for nutrients to encourage. The scoring system ranges from −15 to 40 points, with a lower score reflecting a better nutritional quality.

A method similar to the above-described method for the HSR was used to estimate FVNL points according to the Nutri-Score guidelines for each item in the R24W database. Of note, the Nutri-Score guidelines for FVNL points differ slightly from the HSR guidelines, and FVNL content was therefore estimated at ≤40%, >40%, >60%, and >80% and scored 0, 1, 2, or 5 points for foods, as well as 0, 2, 4, or 10 points for beverages, respectively. The eligibility of some ingredients for FVNL points also differs slightly between the Nutri-Score and the HSR. For example, potatoes, spices, chia seeds, and flax seeds count as FVNL ingredients under the HSR, but not the Nutri-Score. Inversely, rapeseed, walnut, and olive oils count as FVNL under the Nutri-Score, but not the HSR [25,30].

Classification into 1 of the 4 Nutri-Score categories, determination of FVNL points, and calculation of the Nutri-Score scores for each item from the R24W database has been completed independently by 2 members of the research team (AC and MT). At each of these steps, results were compared, and discrepancies were discussed whenever necessary in order to reach a consensus.

#### Nutrient-Rich Food Index 6.3

The NRF represents a family of NP models developed for academic purposes by the University of Washington, in the United States [27]. The different versions of the NRF vary in the number of beneficial nutrients included in their algorithm, ranging from 6 (NRF 6.3) to 15 (NRF 15.3), with the NRF 9.3 being the most widely used version. The NRF 6.3 has, however, been selected for this study because some nutrients included in version 9.3 are not readily available on the Canadian NFt (e.g., potassium, magnesium, and vitamin E). Therefore, it would not allow its use as part of future work on the characterization of the food supply’s nutritional quality in Québec to be conducted by the Observatory [24]. In the NRF 6.3, the calculated summary score is presented as the sum of percent daily values (DVs) from 6 nutrients to encourage (proteins, fiber, vitamin A, vitamin C, calcium, and iron), subtracted from the sum of percent DVs from 3 nutrients to limit (saturated fatty acids, added sugars, and sodium) [27]. Because data on added sugars (i.e., sugars or syrups added to foods or beverages during processing, preparation or at the table) [33] are not readily available in Canadian food databases, total sugars were used in the original version of the NRF 6.3 to be tested, with 100% DV set to 100 g as per Health Canada’s Table of DVs for nutrition labeling [34]. Calculations were based on a food item’s nutrient values per 100 kcal, and each nutrient could not exceed 100% of its DV to avoid overvaluing foods that provide very large amounts of some specific nutrients. The final score attributed to a food item by the NRF 6.3 theoretically ranges from −300 to 600 points, with a higher score reflecting a better nutritional quality.

Calculation of the NRF scores for each item from the R24W database has been completed independently by 2 members of the research team (AC and MT). Results were compared, and discrepancies were discussed whenever necessary in order to reach a consensus. Supplementary Table 1 presents a summary of the algorithm of each NP model.

#### Modification of NP model algorithms to include free sugars

Modified versions of the HSR system, Nutri-Score, and NRF 6.3, which consisted of the same algorithm for each NP model apart from the total sugars component being replaced by free sugars, have been created to fulfill the study’s second objective. For the HSR system, the thresholds for total sugars started at 3.75% of the DV of 100 g for total sugars and increased by the same percentage for each threshold [35]. Thus, the thresholds for the modified version started at 3.75% of the DV for free sugars (i.e., 50 g, which corresponds to the WHO guidelines on free sugars) [36] and increased by the same percentage for every threshold. The same modified thresholds were used for both foods and beverages (Supplementary Table 2). In the original version of the Nutri-Score, the total sugars thresholds for the beverages category were already 3 times smaller than the thresholds for the foods category. Indeed, the total sugars thresholds for beverages in the original version were already stricter than those for foods, as a way to better reflect PNNS recommendations to limit the intake of sugars [32]. Because the Nutri-Score guidelines exclude dairy beverages from the beverages category and rather consider them as foods, it can be assumed that the total sugar content of all other beverages reflects the free sugar content. Therefore, thresholds for beverages were not adjusted in the modified version of the Nutri-Score because they already took free sugars into account. In fact, the stricter thresholds for beverages were now also applied to foods in the modified version of the Nutri-Score (Supplementary Table 2). Modifications to the NRF 6.3 consisted of replacing the DV of 100 g for total sugars used in the equation (as previously described) by a DV of 50 g for free sugars.

Application of each of the 3 modified NP models in the R24W database required data on free sugars to be available for each food item. Of note, the free sugar content of each item had already been estimated as part of previous work using a step-by-step approach published by Bergeron et al. [22], which was adapted from an algorithm by Bernstein et al. [37].

#### Calculation of NP-derived individual scores representing the nutritional quality of foods consumed

An NP score has first been calculated for the 2670 items in the R24W database using each of the 3 tested NP models, in both their original and modified versions (i.e., including total sugars vs free sugars, respectively). Only 2656 items were considered in the analyses because alcoholic beverages (*n* = 14) are not covered by most NP models and were therefore excluded. Then, for each version of the 3 tested NP models, an “energy-weighted, food-based score” at the individual level has been calculated for each dietary collection day as done in previous research [7,27,38]. The following equation was used:

$$\text{Energy}-\text{weighted,}\;\text{food}-\text{based}\;\text{score}\;\text{from}\;a\;\text{single}\;24-h\;\text{recall}=\frac{\sum_{i=1}^{n}{\text{NPS}}_{i}\text{x }E_{i}}{\sum_{i=1}^{n}E_{i}}$$

where NPS_i_ represents the item’s NP score, *E*_*i*_ represents the amount of energy (kilocalories) provided by the item on a given day, and *n* represents the total number of different items consumed. Briefly, the energy-weighted, food-based score was obtained by calculating the sum of the energy supplied by every item consumed by an individual during a given day multiplied by its NP score and then divided by the individual’s total energy intake on that day (excluding energy from alcoholic beverages). The final energy-weighted, food-based score for each individual was then calculated as an average of the scores from 3 (*n* = 1008) or 2 (*n* = 11) 24-h recalls, provided that only the participants with data available for ≥2 recalls would be included in the analyses.

#### Diet quality measure used for convergent validity testing: the Healthy Eating Food Index 2019

The Healthy Eating Food Index (HEFI) 2019 is a validated Canadian-specific diet quality index developed to measure the alignment of eating patterns with Canada’s Food Guide 2019 recommendations on food choices among Canadians aged 2 y and older [39,40]. Briefly, it considers 10 components for a maximum of 80 points: 5 components related to foods (vegetables and fruits, 20 points; whole-grain foods, 5 points; grain food ratio, 5 points; protein foods, 5 points; and plant-based protein foods, 5 points), 1 component consisting of beverages (10 points), and 4 components related to nutrients (fatty acids ratio, 5 points; saturated fats, 5 points; free sugars, 10 points; and sodium, 10 points). All components are expressed as ratios. The HEFI-2019 is fully adapted for use based on dietary intake data derived from 24-h recalls. A HEFI-2019 score was calculated for each completed 24-h recall, and the mean HEFI-2019 score from all recalls of a given participant was used in the analyses.

#### Health status data used for criterion-related validity testing: biomarkers of cardiometabolic risk and presence of metabolic syndrome

As previously described elsewhere [12,17,22], body weight, waist circumference, body fat percentage, and systolic and diastolic blood pressure were measured according to a standardized protocol [12,41]. Briefly, body weight and fat were measured using the body composition analyzer BC-418 (Tanita). Waist circumference as well as systolic and diastolic blood pressure were determined as the mean of 3 measurements. Waist circumference was measured with a tape at the end of a normal expiration between the last rib and the top of the iliac crest. Blood pressure measurements were taken 3 min apart in the sitting position after a seated 10-min rest with an automated blood pressure monitor (Digital BPM HEM-907XL model; Omron). Collected blood samples were analyzed for blood lipid profile [total, LDL, and HDL cholesterol, and triglycerides (TGs)], glucose homeostasis (fasting glucose, insulin, and HOMA-IR) and inflammatory markers [C-reactive protein (CRP)] as previously described elsewhere [12,41]. Briefly, blood lipids were assessed with the use a Roche Modular P system (Roche Diagnostics, Manheim, Germany) and the Friedewald equation. Fasting blood glucose and insulin concentrations were examined with colorimetry (Hexokinase Method, Roche Modular P System) and electrochemiluminescence (Cobas 6000, Roche Diagnostics), respectively. HOMA-IR was then calculated using fasting insulin and glucose concentrations with the method described by Matthews et al. [42]. Commercial ELISA kits were used to measure CRP levels (Biocheck, Foster City, CA). Adiponectin concentrations were specifically measured in the context of the current study using a commercial ELISA kit (As One International, Inc.).

The presence of metabolic syndrome (MetS) was determined based on the Joint Interim Statement from the International Diabetes Federation Task Force on Epidemiology and Prevention; National Heart, Lung, and Blood Institute; American Heart Association; World Heart Federation; International Atherosclerosis Society; and International Association for the Study of Obesity [43]. This definition was used because it was the most recent in the literature, took ethnicity into account in the assessment of waist circumference, and required to have ≥3 of the following criteria (later referred to as MetS traits) to establish the diagnosis of MetS: *1*) elevated waist circumference (depending on ethnic groups, ≥80 to ≥90 cm for females, and ≥90 to ≥102 cm for men); *2*) elevated TGs (≥1.7 mmol/L or drug treatment for this condition); *3*) low HDL cholesterol (<1.3 mmol/L for females, <1.0 mmol/L for men, or drug treatment for this condition); *4*) elevated blood pressure (systolic ≥130 mm Hg and/or diastolic ≥85 mm Hg or antihypertensive drug treatment); *5*) elevated fasting glucose (≥100 mg/dL or drug treatment for this condition). Participants meeting 3 or more of these criteria were classified as having MetS. Regarding MetS traits, 114 participants reported taking medication for elevated blood pressure, 97 participants for elevated TGs, and 39 participants for diabetes.

### Statistical analyses

Statistical analyses were performed using SAS OnDemand for Academics [44]. Participant characteristics were first reported for the overall sample. Mean NP-derived scores for the 3 original NP models were also reported across participant characteristics using least square means to determine the presence of differences between characteristic subgroups.

As part of objective 1, in the context of convergent validity testing of the original NP models (i.e., including total sugars), associations between NP-derived individual scores and the HEFI-2019 were examined using multivariable general linear models with *PROC SURVEYREG*. Given that the final PREDISE sample was slightly larger than what was planned originally, sampling weights were used to ensure exact age and sex-representativeness in each region [12]. The percentage of variation (adjusted R^2^) in the diet quality index (i.e., HEFI-2019) explained by each NP model and their covariates (the latter described below) was also calculated, as done in previous research [27]. In the context of criterion-related validity testing, associations between NP-derived individual scores and cardiometabolic risk factors [BMI (in kg/m^2^); waist circumference; body fat percentage; systolic and diastolic blood pressure; concentrations of TGs; glucose; insulin; total, LDL, and HDL cholesterol; and inflammatory biomarkers] were also examined using multivariable general linear models with *PROC SURVEYREG*. Because of a few missing or excluded values, the number of participants included in these analyses varied slightly across each cardiometabolic risk factor (*n* = 942–1005) (Figure 1). Reasons for missing cardiometabolic risk factors included: loss of consciousness, unable to perform the blood sample, or unspecified. For CRP specifically, participants with a CRP value ≥10 mg/L (*n* = 63) were excluded as it may indicate acute inflammation. Normal probability plots were used to visually assess normality of the residuals. Seven variables had to be log-transformed because the residuals did not follow a normal distribution (HOMA-IR, HDL-C, TG, glucose, insulin, CRP, and adiponectin). Additionally, associations of NP-derived individual scores with MetS and MetS traits were examined using logistic regression analysis. Missing values for cardiometabolic risk factors or MetS traits were not imputed, because they are the dependent variables in the analyses.

Covariates in the analyses were determined by examining the presence of statistically significant differences between the different participant characteristics subgroups for each NP model. If a difference was detected for a given characteristic with ≥1 NP model, it was decided to include the variable as a covariate. All models were therefore adjusted for sex, age (continuous), administrative region (*Estrie*, *Saguenay-Lac-Saint-Jean*, *Capitale-Nationale*/*Chaudière-Appalaches*, *Montréal*, or *Mauricie*), educational level (high school or no diploma, *Cégep*, or university), ethnicity (Caucasian, African/African American, Hispanic, or other), BMI (continuous, kg/m^2^; except when BMI was analyzed), smoking status (yes, formerly, or never), alcohol intake (continuous, g/d), and reporting status (underreporter, plausible, or overreporter of total energy intake). Reporting status was assessed by the method of Huang et al. [45], comparing reported energy intakes with predicted energy requirements. Underreporters had a reported energy intake-to-predicted energy requirement ratio of <0.78 and overreporters had a ratio >1.22. The Food Quality Observatory’s statistician was consulted to confirm that the sample size was sufficient to adjust for all covariates we had identified. Because our primary goal was to compare results between NP models, without necessarily generalizing the observed associations to the entire population of Québec, we decided to not impute missing values for any of the covariates. Finally, another model additionally including physical activity as a covariate was also tested, but due to a high number of missing values for this variable (*n* = 119) and given that adjusting for this variable led to similar results, it was not retained in the final analyses. In this model, physical activity levels (low, moderate, and high) were assessed based on the Guidelines for Data Processing and Analysis of the International Physical Activity Questionnaire [46].

The same types of analyses as in objective 1 were conducted for objective 2, however, using the modified version of each NP model (i.e., including free sugars) instead of their original version. Associations at *P* < 0.05 were considered statistically significant. All analyses conducted for both convergent and criterion-related validity testing were prespecified.

## Results

### Participant characteristics

Table 1 [46,47] summarizes the main participant characteristics (*N* = 1019). Sex distribution (male/female) was relatively equal within the study sample. Most participants lived in the urban regions of *Capitale-Nationale/Chaudière-Appalaches* or *Montréal*, held a postsecondary degree, were Caucasian, nonsmokers, had a moderate or high physical activity level, and had a low risk alcohol intake. Slightly less than a quarter of participants were affected by MetS.

**TABLE 1:** Summary of the participant characteristics (*N* = 1019).^1^

Characteristic	Value
Sex	
Female	516 (51)
Male	503 (49)
Age (y)	
18–34	351 (34)
35–49	305 (30)
50–65	363 (36)
BMI group (kg/m^2^)	
Normal (<25.0)	405 (40)
Overweight (25.0–29.9)	336 (33)
Obese (≥30.0)	276 (27)
Administrative region	
Capitale-Nationale/Chaudière-Appalaches	381 (37)
Estrie	109 (11)
Mauricie	91 (9)
Montréal	341 (33)
Saguenay-Lac Saint-Jean	97 (10)
Education	
High school or no diploma	242 (24)
Cégep^2^	311 (31)
University	460 (45)
Household income ($CAD)	
<30,000	146 (16)
≥30,000 to <60,000	264 (29)
≥60,000 to <90,000	182 (20)
≥90,000	333 (36)
Ethnicity	
Caucasian	940 (93)
African/African American	20 (2)
Hispanic	16 (2)
Other	34 (3)
Smoking status	
Yes	128 (13)
Formerly	336 (33)
Never	555 (54)
Reporting status	
Underreporter (rEI:pER ≤ 0.78)	123 (12)
Plausible reporter (0.78 < rEI:pER < 1.22)	637 (63)
Overreporter (rEI:pER ≥ 1.22)	259 (25)
Physical activity level^3^	
Low	174 (19)
Moderate	349 (39)
High	377 (42)
Alcohol intake^4^	
Low risk	895 (88)
High risk	124 (12)
Metabolic syndrome	
Yes	235 (23)
No	772 (77)

Values are *n* (%).

Abbreviations: Cégep, *collège d’enseignement général et professionnel*; pER, predicted energy requirement; rEI, self-reported energy intake.

1 Rounding of the percentages might have caused the sample size to equal 100% ± 1%. There were missing values for BMI (*n* = 2), education (*n* = 6), household income (*n* = 94), ethnicity (*n* = 9), physical activity (*n* = 119), and metabolic syndrome (*n* = 12).

2 Cégep is a preuniversity and technical college institution specific to the Québec educational system.

3 Physical activity categories based on International Physical Activity Questionnaire scoring [46].

4 According to Canada’s alcohol drinking guidelines [47], low risk consumption refers to 2 standard drinks per day or less for females and 3 standard drinks per day or less for men, which is the equivalent of ≤26.9 and ≤40.35 g of alcohol/d, respectively.

Table 2 presents mean NP-derived scores for the original version of each NP model across participant characteristics. For all 3 original NP models, a better quality of foods consumed, as determined by a lower NP-derived individual score for HSR and Nutri-Score but a higher NP-derived individual score for NRF 6.3, was associated with having a lower BMI (all *P* ≤ 0.0004), being a nonsmoker (both former or never smoker; all *P* ≤ 0.008), and being an underreporter (all *P* ≤ 0.0001). For the HSR specifically, a better quality of foods consumed was also associated with living in the area of Montréal when compared with Saguenay-Lac Saint-Jean (*P* = 0.005) and being from an African or African American ethnicity when compared with Caucasian (*P* = 0.0006). For both the Nutri-Score and the NRF 6.3, a better quality of foods consumed was also associated with being of the female sex (*P* ≤ 0.006), being older (*P* = 0.02), and having higher education and physical activity levels (*P* ≤ 0.03). For the Nutri-Score specifically, a better quality of foods consumed was further associated with living in the area of Montréal (*P* < 0.0001). Finally, for the NRF 6.3 specifically, a better quality of foods consumed was further associated with having a lower alcohol intake (*P* = 0.005).

**TABLE 2:** Mean nutrient profiling–derived individual scores for the 3 original nutrient profiling models across participant characteristics.^1^

Characteristic (*N* = 1019)	Original versions of nutrient profiling models^2^					
	Health Star Rating		Nutri-Score		Nutrient-Rich Food 6.3	
	Values	*P*	Values	*P*	Values	*P*
Sex		0.57		0.006		<0.0001
Female (*n* = 516)	5.39 ± 0.13^a^		6.26 ± 0.10^a^		14.60 ± 0.31^a^	
Male (*n* = 503)	5.50 ± 0.14^a^		6.67 ± 0.11^b^		12.44 ± 0.32^b^	
Age (y)		0.95		0.02		0.02
18–34 (*n* = 351)	5.48 ±0.16^a^		6.73 ± 0.13^a^		12.74 ± 0.39^a^	
35–49 (*n* = 305)	5.45 ± 0.17^a^		6.47 ± 0.14^a,b^		13.50 ± 0.40^a,b^	
50–65 (*n* = 363)	5.40 ± 0.17^a^		6.22 ± 0.12^b^		14.28 ± 0.37^b^	
BMI group (kg/m^2^)		0.0003		0.0004		0.0004
Normal (<25.0) (*n* = 405)	5.02 ± 0.15^a^		6.15 ± 0.12^a^		14.51 ± 0.36^a^	
Overweight (25.0–29.9) (*n* = 336)	5.50 ± 0.17^a,b^		6.46 ± 0.13^a,b^		13.43 ± 0.38^a,b^	
Obese (≥30.0) (*n* = 276)	5.99 ± 0.19^b^		6.90 ± 0.15^b^		12.30 ± 0.42^b^	
Administrative region		0.005		<0.0001		0.18
Capitale-Nationale/Chaudière-Appalaches (*n* = 381)	5.45 ± 0.16^a,b^		6.50 ± 0.12^a^		13.53 ± 0.35^a,b^	
Estrie (*n* = 109)	5.62 ± 0.31^a,b^		6.77 ± 0.24^a,b^		13.54 ± 0.72^a,b^	
Mauricie (*n* = 91)	5.87 ± 0.35^a,b^		6.86 ± 0.24^a,b^		13.11 ± 0.92^a,b^	
Montréal (*n* = 341)	5.01 ± 0.17^a^		5.98 ± 0.13^c^		14.07 ± 0.38^a^	
Saguenay-Lac Saint-Jean (*n* = 97)	6.26 ± 0.32^b^		7.24 ± 0.23^b^		12.24 ± 0.64^b^	
Education		0.12		0.01		0.001
High school or no diploma (*n* = 242)	5.62 ± 0.22^a^		6.68 ± 0.16^a^		12.42 ± 0.49^a^	
Cégep^3^ (*n* = 311)	5.63 ± 0.18^a^		6.66 ± 0.14^a^		13.11 ± 0.40^a^	
University (*n* = 460)	5.22 ± 0.14^a^		6.22 ± 0.11^b^		14.44 ± 0.32^b^	
Household income ($CAD)		0.90		0.52		0.91
<30,000 (*n* = 146)	5.46 ± 0.32^a^		6.57 ± 0.23^a^		13.68 ± 0.74^a^	
≥30,000 to <60,000 (*n* = 264)	5.56 ± 0.20^a^		6.50 ± 0.16^a^		13.77 ± 0.47^a^	
≥60,000 to <90,000 (*n* = 182)	5.33 ± 0.23^a^		6.27 ± 0.17^a^		13.36 ± 0.46^a^	
≥90,000 (*n* = 333)	5.51 ± 0.15^a^		6.58 ± 0.12^a^		13.75 ± 0.36^a^	
Ethnicity		0.0006		0.003		0.37
Caucasian (*n* = 940)	5.56 ± 0.10^a^		6.55 ± 0.08^a^		13.46 ± 0.23^a^	
African/African American (*n* = 20)	3.71 ± 0.65^b^		5.36 ± 0.48^a^		14.91 ± 1.17^a^	
Hispanic (*n* = 16)	3.59 ± 0.76^a,b^		4.94 ± 0.64^a^		15.46 ± 1.52^a^	
Other (*n* = 34)	4.53 ± 0.50^a,b^		5.89 ± 0.39^a^		13.25 ± 1.22^a^	
Smoking status		0.004		0.008		<0.0001
Yes (*n* = 128)	6.32 ± 0.29^a^		7.07 ± 0.22^a^		10.34 ± 0.60^a^	
Formerly (*n* = 336)	5.39 ± 0.18^b^		6.28 ± 0.13^b^		14.09 ± 0.39^b^	
Never (*n* = 555)	5.27 ± 0.12^b^		6.44 ± 0.10^b^		13.93 ± 0.30^b^	
Reporting status		<0.0001		<0.0001		0.0001
Underreporter (rEI:pER ≤ 0.78) (*n* = 123)	4.13 ± 0.26^a^		5.47 ± 0.20^a^		15.82 ± 0.71^a^	
Plausible reporter (0.78 < rEI:pER < 1.22) (*n* = 637)	5.35 ± 0.12^b^		6.43 ± 0.09^b^		13.59 ± 0.28^b^	
Overreporter (rEI:pER ≥ 1.22) (*n* = 259)	6.29 ± 0.22^c^		7.02 ± 0.16^c^		12.36 ± 0.41^c^	
Physical activity level^4^		0.07		0.03		0.01
Low (*n* = 174)	5.73 ± 0.26^a^		6.72 ± 0.19^a^		12.92 ± 0.50^a^	
Moderate (*n* = 349)	5.51 ± 0.15^a^		6.51 ± 0.12^a,b^		13.38 ± 0.36^a,b^	
High (*n* = 377)	5.11 ± 0.16^a^		6.18 ± 0.12^b^		14.60 ± 0.38^b^	
Alcohol intake^5^		0.08		0.45		0.005
Low risk (*n* = 895)	5.38 ± 0.11^a^		6.44 ± 0.08^a^		13.73 ± 0.24^a^	
High risk (*n* = 124)	5.88 ± 0.27^a^		6.61 ± 0.20^a^		12.15 ± 0.51^b^	

Abbreviations: Cégep, *collège d’enseignement général et professionnel*; pER, predicted energy requirements; rEI, self-reported energy intake.

1 Values are means ± SEs. *P* values were determined using least square means. Subgroups without a common superscript letter are statistically different (Tukey-Kramer; *P* < 0.05). There were missing values for BMI (*n* = 2), education (*n* = 6), household income (*n* = 94), ethnicity (*n* = 9), physical activity (*n* = 119), and metabolic syndrome (*n* = 12).

2 For the Health Star Rating and the Nutri-Score, a lower score indicates a better quality of foods consumed, whereas a higher score indicates a better quality of foods consumed for the Nutrient-Rich Food 6.3.

3 Cégep is a preuniversity and technical college institution specific to the Québec educational system.

4 Physical activity categories based on International Physical Activity Questionnaire scoring [46].

5 According to Canada’s alcohol drinking guidelines [47], low risk consumption refers to 2 standard drinks per day for females and 3 standard drinks per day for men, which is the equivalent of 26.9 and 40.35 g of alcohol/d, respectively.

### Convergent and criterion-related validity testing of the original versions of NP models

Results from the convergent validity testing of the original NP models against the HEFI-2019 showed that for all 3 models, a better quality of foods consumed was associated with a higher HEFI-2019 score (Table 3) (all *P* < 0.0001). Only a slightly higher percentage of variation in the HEFI-2019 was explained by the Nutri-Score (adjusted *R*^2^ = 0.55) than that by the HSR (adjusted *R*^2^ = 0.49) and NRF 6.3 (adjusted *R*^2^ = 0.43).

**TABLE 3:** Linear regression between nutrient profiling–derived individual scores for the 3 original nutrient profiling models and HEFI-2019 as well as biomarkers of cardiometabolic risk.^1^

	Original version of nutrient profiling models^2^														
	Health Star Rating					Nutri-Score					Nutrient-Rich Food 6.3				
	β	SD	*P*	STB	Adjusted *R*^2^	β	SD	*P*	STB	Adjusted *R*^2^	β	SD	*P*	STB	Adjusted *R*^2^
HEFI-2019	−2.02	0.085	<0.0001	−0.64	0.49	−2.87	0.094	<0.0001	−0.70	0.55	0.81	0.039	<0.0001	0.59	0.43
BMI^3^ (kg/m^2^)	0.36	0.061	<0.0001	0.18	0.12	0.48	0.082	<0.0001	0.19	0.12	−0.16	0.025	<0.0001	−0.18	0.12
Waist circumference (cm)	0.12	0.061	0.04	0.02	0.89	0.18	0.078	0.02	0.03	0.89	−0.04	0.025	0.09	−0.02	0.89
Body fat (%)	0.06	0.040	0.12	0.02	0.86	0.08	0.055	0.13	0.02	0.86	−0.04	0.019	0.048	−0.03	0.86
DBP (mm Hg)	0.24	0.100	0.02	0.07	0.29	0.30	0.129	0.02	0.07	0.29	−0.08	0.040	0.04	−0.06	0.29
SBP (mm Hg)	0.14	0.127	0.26	0.03	0.30	0.14	0.168	0.40	0.02	0.30	−0.04	0.054	0.50	−0.02	0.30
Total cholesterol (mmol/L)	0.01	0.011	0.37	0.03	0.08	0.01	0.014	0.37	0.03	0.08	−0.002	0.005	0.75	−0.01	0.08
LDL cholesterol (mmol/L)	0.004	0.010	0.68	0.01	0.04	0.01	0.013	0.48	0.02	0.04	0.002	0.004	0.60	0.02	0.04
HDL cholesterol^4^ (mmol/L)	−0.005	0.003	0.09	−0.05	0.15	−0.01	0.004	0.01	−0.08	0.15	0.002	0.001	0.19	0.04	0.14
TG^4^ (mmol/L)	0.02	0.005	0.001	0.10	0.16	0.02	0.007	0.002	0.10	0.16	−0.01	0.002	0.002	−0.09	0.16
Fasting glucose^4^ (mmol/L)	0.002	0.001	0.15	0.04	0.15	0.001	0.002	0.76	0.01	0.14	−0.0002	0.0006	0.77	−0.01	0.14
Fasting insulin^4^ (pmol/L)	0.01	0.005	0.01	0.08	0.22	0.02	0.006	0.047	0.09	0.23	−0.003	0.002	0.17	−0.04	0.22
HOMA-IR^4^	0.01	0.005	0.01	0.08	0.25	0.02	0.007	0.01	0.08	0.25	−0.003	0.002	0.21	−0.04	0.24
CRP^4^ (mg/mL)	0.009	0.009	0.36	0.03	0.28	0.02	0.012	0.11	0.05	0.29	−0.01	0.004	0.07	−0.05	0.29
Adiponectin^4^ (ng/mL)	−0.004	0.004	0.43	−0.02	0.26	−0.01	0.006	0.35	−0.03	0.26	0.001	0.002	0.78	0.01	0.26

Abbreviations: CRP, C-reactive protein; DBP, diastolic blood pressure; HEFI, Healthy Eating Food Index; SBP, systolic blood pressure; STB, standardized β coefficient; TG, triglycerides.

1 Multivariable linear models provide regression coefficients (β or STB) for outcome variables for a 1-point increase in nutrient profiling–derived scores adjusted for sex, age, administrative region, education level, ethnicity, BMI, smoking status, reporting status, and alcohol intake. The number of participants with data available for the analyses differed depending on the outcome: HEFI-2019 and BMI (*n* = 1005); waist circumference, DBP, and SBP (*n* = 1004); body fat (*n* = 1002); total cholesterol, LDL cholesterol, HDL cholesterol, TG, and fasting glucose (*n* = 995); fasting insulin and HOMA-IR (*n* = 993); CRP (*n* = 942); and adiponectin (*n* = 1001).

2 For the Health Star Rating and the Nutri-Score, a lower score indicates a better quality of foods consumed, whereas a higher score indicates a better quality of foods consumed for the Nutrient-Rich Food 6.3.

3 Not adjusted for BMI.

4 For HDL cholesterol, TG, glucose, insulin, HOMA-IR, CRP, and adiponectin, analyses were performed on log-transformed data.

In terms of criterion-related validity testing of the original NP models against biomarkers of cardiometabolic risk, for all 3 models, a higher quality of foods consumed was associated with a lower BMI (Table 3) [β: between −0.16 kg/m^2^ (NRF 6.3) and 0.48 kg/m^2^ (Nutri-Score), all *P* < 0.0001], a lower diastolic blood pressure [β: between −0.08 mm Hg (NRF 6.3) and 0.30 mm Hg (Nutri-Score); all *P* ≤ 0.04], and lower TGs [β: between −0.01 mmol/L (NRF 6.3) and 0.02 mmol/L (HSR and Nutri-Score); all *P* ≤ 0.002]. A better quality of foods consumed as determined by the HSR and the Nutri-Score was additionally associated with a lower waist circumference (β: 0.12 and 0.18 cm, respectively; both *P* ≤ 0.04), a lower fasting insulin (β: 0.01 and 0.02 pmol/L, respectively; both *P* ≤ 0.047), and a lower HOMA-IR (β: 0.01 and 0.02, respectively; both *P* = 0.01). A higher quality of foods consumed was associated with higher HDL cholesterol for the Nutri-Score only (β: −0.01 mmol/L; *P* = 0.01), as well as with lower body fat percentage for the NRF 6.3 only (β: −0.04%; *P* = 0.048).

Table 4 shows the results of further criterion-related validity testing by calculating the odds of having MetS or MetS traits for each of the original versions of NP models. A lower quality of foods consumed, as determined by the HSR and the Nutri-Score, was associated with higher odds of having MetS (OR: 1.064; 95% CI: 1.002, 1.130, and OR: 1.085; 95% CI: 1.001, 1.175, respectively; *P* ≤ 0.046), although no association was observed with the NRF 6.3 (OR: 0.987; 95% CI: 0.961, 1.014; *P* = 0.33). Regarding the odds of presenting MetS traits, for the HSR only, a lower quality of foods consumed was associated with higher odds of having elevated blood pressure and fasting glucose (both OR: 1.06; *P* ≤ 0.045). For the NRF 6.3, specifically, a lower quality of foods consumed was associated with higher odds of having elevated TGs (*P* = 0.005).

**TABLE 4:** Odds ratios between the nutrient profiling–derived individual scores for the original versions of nutrient profiling models and metabolic syndrome outcomes.^1^

	Original version of nutrient profiling models^2^		
	Health Star Rating	Nutri-Score	Nutrient-Rich Food 6.3
Metabolic syndrome	1.064 (1.002, 1.130)	1.085 (1.001, 1.175)	0.987 (0.961, 1.014)
*P*	0.04	0.046	0.33
Elevated waist circumference	1.000 (0.919, 1.089)	1.008 (0.900, 1.130)	1.010 (0.973, 1.049)
*P*	0.99	0.89	0.61
Elevated TG	1.048 (0.995, 1.105)	1.067 (0.996, 1.143)	0.966 (0.943, 0.990)
*P*	0.08	0.07	0.005
Low HDL cholesterol	1.028 (0.976, 1.083)	1.054 (0.985, 1.128)	0.997 (0.975, 1.020)
*P*	0.30	0.13	0.82
Elevated blood pressure	1.064 (1.008, 1.123)	1.072 (0.998, 1.152)	0.991 (0.967, 1.015)
*P*	0.03	0.06	0.45
Elevated fasting glucose	1.059 (1.001, 1.121)	1.054 (0.978, 1.135)	0.987 (0.962, 1.013)
*P*	0.045	0.17	0.32

Abbreviations: TG, triglyceride.

1 Values are ORs (95% CIs) estimated through logistic regression adjusted for sex, age, administrative region, education level, ethnicity, BMI, smoking status, reporting status, and alcohol intake. The number of participants with data available for the analyses differed depending on the outcome: metabolic syndrome, low HDL cholesterol, and elevated blood pressure (*n* = 995); elevated waist circumference (*n* = 1004); elevated TG (*n* = 987); and elevated fasting glucose (*n* = 988).

2 For the Health Star Rating and the Nutri-Score, a lower score indicates a better quality of foods consumed, whereas a higher score indicates a better quality of foods consumed for the Nutrient-Rich Food 6.3.

### Convergent and criterion-related validity testing of the modified versions of NP models

**FIGURE 2:**
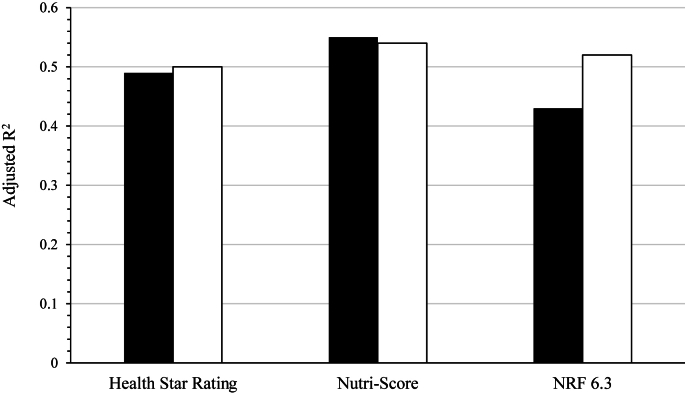
Adjusted *R*^2^ from the linear regression between NP-derived individual scores and the Healthy Eating Food Index-2019 for the original (with total sugars; black bars) and modified (with free sugars; white bars) versions of each NP model. NP, nutrient profiling; NRF, Nutrient-Rich Food.

Results from the convergent and criterion-related validity testing of the modified NP models, namely including free sugars as opposed to total sugars in their algorithm, are presented in TABLE 5, TABLE 6 (see Figure 2 and Supplementary Tables 3–5 for a comparison of both versions of a given NP model within the same figure or table). In terms of convergent validity testing, very similar to results observed with the original models, consuming foods of higher nutritional quality as determined by each of the 3 modified NP models was associated with a higher HEFI-2019 score (Figure 2 and Table 5) [adjusted *R*^2^ varying from 0.53 (Nutri-Score) to 0.50 (HSR); all *P* < 0.0001].

**TABLE 5:** Linear regression between nutrient profiling–derived individual scores for the 3 modified nutrient profiling models and HEFI-2019 as well as biomarkers of cardiometabolic risk.^1^

	Modified version of nutrient profiling models^2^														
	Health Star Rating					Nutri-Score					Nutrient-Rich Food 6.3				
	β	SD	*P*	STB	Adjusted *R*^2^	β	SD	*P*	STB	Adjusted *R*^2^	β	SD	*P*	STB	Adjusted *R*^2^
HEFI-2019	−2.00	0.084	<0.0001	−0.64	0.50	−2.56	0.085	<0.0001	−0.69	0.53	0.82	0.031	<0.0001	0.67	0.52
BMI^3^ (kg/m^2^)	0.35	0.060	<0.0001	0.18	0.12	0.42	0.074	<0.0001	0.18	0.12	−0.14	0.023	<0.0001	−0.19	0.12
Waist circumference (cm)	0.11	0.060	0.07	0.02	0.89	0.20	0.070	0.004	0.03	0.89	−0.05	0.022	0.03	−0.02	0.89
Body fat (%)	0.06	0.040	0.12	0.02	0.86	0.09	0.049	0.06	0.02	0.86	−0.04	0.017	0.03	−0.03	0.86
DBP (mm Hg)	0.22	0.099	0.02	0.07	0.29	0.27	0.115	0.02	0.07	0.29	−0.08	0.036	0.02	−0.07	0.29
SBP (mm Hg)	0.12	0.127	0.33	0.03	0.30	0.12	0.155	0.44	0.02	0.30	−0.04	0.048	0.42	−0.02	0.30
Total cholesterol (mmol/L)	0.01	0.011	0.35	0.03	0.08	0.01	0.013	0.41	0.03	0.08	−0.003	0.004	0.52	−0.02	0.08
LDL cholesterol (mmol/L)	0.004	0.010	0.65	0.02	0.04	0.01	0.012	0.51	0.02	0.04	0.0003	0.004	0.93	0.003	0.04
HDL cholesterol^4^ (mmol/L)	−0.004	0.003	0.15	−0.04	0.14	−0.01	0.003	0.005	−0.09	0.15	0.002	0.001	0.08	0.05	0.15
TG^4^ (mmol/L)	0.01	0.005	0.003	0.09	0.16	0.02	0.006	0.001	0.10	0.16	−0.01	0.002	0.001	−0.10	0.16
Fasting glucose^4^ (mmol/L)	0.002	0.001	0.18	0.04	0.15	0.001	0.002	0.74	0.01	0.14	0.00001	0.0005	0.99	0.001	0.14
Fasting insulin^4^ (pmol/L)	0.01	0.005	0.03	0.07	0.22	0.02	0.006	0.004	0.09	0.23	−0.003	0.002	0.06	−0.05	0.22
HOMA-IR^4^	0.01	0.005	0.02	0.07	0.25	0.02	0.006	0.009	0.08	0.25	−0.003	0.002	0.11	−0.05	0.25
CRP^4^ (mg/mL)	0.01	0.009	0.39	0.02	0.28	0.02	0.011	0.04	0.06	0.29	−0.01	0.004	0.03	−0.06	0.29
Adiponectin^4^ (ng/mL)	−0.003	0.004	0.46	−0.02	0.26	−0.005	0.005	0.32	−0.03	0.26	0.001	0.002	0.76	0.01	0.26

CRP, C-reactive protein; DBP, diastolic blood pressure; HEFI, Healthy Eating Food Index; SBP, systolic blood pressure; STB, standardized β coefficient; TG, triglyceride.

1 Multivariable linear models provide regression coefficients (β or STB) for outcome variables for a 1-point increase in nutrient profiling–derived scores adjusted for sex, age, administrative region, education level, ethnicity, BMI, smoking status, reporting status, and alcohol intake. The number of participants with data available for the analyses differed depending on the outcome: HEFI-2019 and BMI (*n* = 1005); waist circumference, DBP, and SBP (*n* = 1004); body fat (*n* = 1002); total cholesterol, LDL cholesterol, HDL cholesterol, TG, and fasting glucose (*n* = 995); fasting insulin and HOMA-IR (*n* = 993); CRP (*n* = 942); and adiponectin (*n* = 1001).

2 For the Health Star Rating and the Nutri-Score, a lower score indicates a better quality of foods consumed, whereas a higher score indicates a better quality of foods consumed for the Nutrient-Rich Food 6.3.

3 Not adjusted for BMI.

4 For HDL cholesterol, TG, glucose, insulin, HOMA-IR, CRP, and adiponectin, analyses were performed on log-transformed data.

**TABLE 6:** Odds ratios between nutrient profiling–derived individual scores for the modified versions of nutrient profiling models and metabolic syndrome outcomes.^1^

	Modified version of nutrient profiling models^2^		
	Health Star Rating	Nutri-Score	Nutrient-Rich Food 6.3
Metabolic syndrome	1.06 (0.995, 1.12)	1.08 (1.004, 1.16)	0.98 (0.96, 1.01)
*P*	0.07	0.04	0.13
Elevated waist circumference	0.999 (0.92, 1.09)	1.01 (0.91, 1.12)	1.01 (0.98, 1.04)
*P*	0.98	0.85	0.60
Elevated TG	1.04 (0.99, 1.10)	1.07 (1.005, 1.14)	0.97 (0.95, 0.99)
*P*	0.14	0.04	0.003
Low HDL cholesterol	1.02 (0.97, 1.08)	1.05 (0.99, 1.12)	0.99 (0.97, 1.01)
*P*	0.43	0.12	0.55
Elevated blood pressure	1.06 (1.004, 1.12)	1.06 (0.996, 1.14)	0.99 (0.97, 1.01)
*P*	0.04	0.06	0.32
Elevated fasting glucose	1.06 (0.998, 1.12)	1.06 (0.989, 1.14)	0.99 (0.97, 1.01)
*P*	0.06	0.10	0.38

TG, triglyceride.

1 Values are ORs (95% CIs) estimated through logistic regression adjusted for sex, age, administrative region, education level, ethnicity, BMI, smoking status, reporting status, and alcohol intake. The number of participants with data available for the analyses differed depending on the outcome: metabolic syndrome, low HDL cholesterol, and elevated blood pressure (*n* = 995); elevated waist circumference (*n* = 1004); elevated TG (*n* = 987); and elevated fasting glucose (*n* = 988).

2 For the Health Star Rating and the Nutri-Score, a lower score indicates a better quality of foods consumed, whereas a higher score indicates a better quality of foods consumed for the Nutrient-Rich Food 6.3.

In terms of criterion-related validity testing, consuming foods of higher nutritional quality as determined by lower individual scores derived from the modified HSR was associated with lower BMI (Table 5) (β: 0.35 kg/m^2^; *P* < 0.0001), lower diastolic blood pressure (β: 0.22 mm Hg; *P* = 0.02), lower concentrations of TGs (β: 0.01 mmol/L; *P* = 0.003), lower fasting insulin (β: 0.01 pmol/L; *P* = 0.03), and lower HOMA-IR score (β: 0.01; *P* = 0.02), all representing associations that were also found with the original version of the HSR. The modified version of the HSR was, however, no longer associated with waist circumference (*P* = 0.07) although showing a similar effect size and percentage of variation explained by the model as in the original version (i.e., β: 0.11 vs 0.12 cm in the modified vs original version; *R*^2^ = 0.89 for both). The modified version of the HSR was also no longer associated with the odds of having MetS and elevated fasting glucose (Table 6) (OR: 1.06; 95% CI: 0.995, 1.12, and OR: 1.06; 95% CI: 0.998, 1.12, respectively) compared with the original version.

Although being associated with BMI, waist circumference, diastolic blood pressure, HDL cholesterol, TGs, fasting insulin, and HOMA-IR (Table 5) (β: between −0.01 mmol/L and 0.42 kg/m^2^, respectively; all *P* ≤ 0.02) as seen with the original Nutri-Score, the modified Nutri-Score was further associated with CRP (β: 0.02 mg/mL; *P* = 0.04). As in the original version, the modified Nutri-Score showed an association with the odds of having MetS (Table 6) (OR: 1.08; 95% CI: 1.004, 1.16; *P* = 0.04). The modified Nutri-Score was, however, further associated with the odds of having elevated TGs (OR: 1.07; 95% CI: 1.005, 1.14; *P* = 0.04).

Finally, similar to its original version, the modified NRF 6.3 was associated with BMI (Table 5) (β: −0.14 kg/m^2^; *P* < 0.0001), body fat percentage (β: −0.04%; *P* = 0.03), diastolic blood pressure (β: −0.08 mm Hg; *P* = 0.02), and TGs (β: −0.01 mmol/L; *P* = 0.001). The modified NRF 6.3 was, however, further associated with waist circumference (β: −0.05 cm; *P* = 0.03) and CRP (β: −0.01 mg/mL; *P* = 0.03). A lower quality of foods consumed, as determined by the modified NRF 6.3, was also associated with higher odds of having elevated TGs as observed with the original version (Table 6) (*P* = 0.003).

## Discussion

To our knowledge, this study was the first to simultaneously test both the convergent and criterion-related validity of multiple NP models—HSR, Nutri-Score, and NRF 6.3—in a French-Canadian sample and to verify whether modified versions of the models, which included free sugars as opposed to total sugars in their algorithm, showed improved validity compared with their original version.

Regarding convergent validity testing, our results show that each of the original NP models are associated with a higher overall diet quality as determined by the HEFI-2019. This may not be completely surprising, given that some components are similar between the tested NP models and the HEFI-2019 (e.g., consideration of saturated fats, sugars, and sodium). The Nutri-Score is the NP model explaining the most variation in the HEFI-2019 (55%), which is 6% more than the HSR and 12% more than the NRF 6.3. Results from our study are consistent with those of previous studies that have tested NP models against other diet quality measures. Different versions of the NRF have indeed been validated against the Dutch Healthy Diet (DHD) index [48] and the Healthy Eating Index (HEI) 2005 [27] in the Dutch and American population, respectively. In both studies, NRF 6.3 was in the top 2 versions of NRF that correlated the most with diet quality. The *R*^2^ between the NRF 6.3 and the HEI-2005 in the study by Fulgoni et al. [27] was the same as the *R*^2^ between the NRF 6.3 and the HEFI-2019 in this study (*R*^2^ = 0.43). Canadian and American dietary guidelines upon which the HEFI-2019 and HEI-2005, respectively, are based on align relatively well, which may explain the similarities in *R*^2^ observed between our study and that of Fulgoni et al. [27]. Interestingly, these *R*^2^ are higher than the 1 observed between the NRF 6.3 and the DHD-index in the study by Sluik et al. [48], at 0.31, suggesting that the NRF 6.3 might not align as much with Dutch dietary guidelines compared with North American guidelines. Regarding the Nutri-Score, Julia et al. [38] validated this model against the PNNS guideline score, a diet quality score that evaluates the level of adherence to French nutritional recommendations. A higher quality of foods consumed as determined by the Nutri-Score was associated with a better overall diet quality. To our knowledge, no study testing the convergent validity of the HSR against a diet quality index was identified in the scientific literature.

In the case of criterion-related validity, our results show that associations of each of the 3 original versions of NP models with a more favorable health status are present, but nevertheless limited. Indeed, the number of associations found between the NP models and the 14 cardiometabolic risk factors varied from 4 (NRF 6.3) to 7 (Nutri-Score). A few other studies have also validated NP models against cardiometabolic risk factors and MetS. Khoury et al. [49] have evaluated a Nutri-Score–derived individual index against 8 cardiovascular disease risk factors at baseline of their prospective study. They observed associations with HDL cholesterol, BMI, and waist circumference. In this study, in addition to these 3 risk factors, associations were found with diastolic blood pressure, TGs, fasting insulin, and HOMA-IR (the last 2 were not evaluated by Khoury et al. [49]). These differences in results could be, in part, due to differences between characteristics of the 2 samples. Indeed, this sample is made up of healthy adults aged between 18 and 65 y, whereas the PREDIMED-plus cohort used in the study by Khoury et al. [49] is rather made up of older adults, aged between 55 and 75 y, and living with overweight/obesity and MetS. Eriksen et al. [50] examined the associations between the UK NP model, which served as the basis for the development of both the Nutri-Score and the HSR, and the following 7 cardiometabolic risk factors: glycated hemoglobin, systolic and diastolic blood pressure, total and HDL cholesterol, waist circumference, and BMI [50]. Their results show that a higher quality of foods consumed as determined by the UK model is associated with lower concentrations of glycated hemoglobin and total cholesterol. However, in contrast with our results, they also show that a higher quality of foods consumed is associated with a higher BMI. The authors reported finding these results challenging because they were somewhat contrary to what was observed in similar studies. However, stratified sensitivity analysis showed an association in the expected direction (higher quality of foods consumed associated with a lower BMI) in both acceptable and underreporters of energy. Another study, by Julia et al. [51], evaluated the predictive validity—a subcategory of criterion-related validity considered as the most robust form of validity testing, as it uses a prospective cohort design as opposed to a cross-sectional design—of an early version of the Nutri-Score against the development of MetS as well as associations with 6 biomarkers included in the definition of MetS. Results from this study show that a higher quality of foods consumed was associated with lower odds of developing MetS (across quartiles of NP scores), as well as with lower diastolic and systolic blood pressure over 13 y. However, a higher quality of foods consumed showed no association with waist circumference, TGs, HDL cholesterol, and fasting glucose over that same period. In sum, results from the current study are quite consistent with those of previous studies, which show a relatively limited number of associations between individual scores derived from NP models and cardiometabolic markers [50,51].

Our study further showed that replacing total sugars by free sugars in the NP models algorithm had little to no effect on the convergent and criterion-related validity testing results as compared with results obtained with the original version of all 3 NP models. No improvement was observed for the HSR, and only slight improvements were observed for the Nutri-Score and the NRF 6.3, mainly in terms of criterion-related validity testing. It is important to note that these differences were very modest considering the similar effect sizes (i.e., β coefficients and *R*^2^) between both versions of each NP model. Only a few studies have studied the replacement of total sugars in NP model algorithms with added or free sugars or vice-versa. In the validation of NRF models against the HEI-2005 [27], all versions of NRF, which took total sugars into account, had a lower *R*^2^, meaning they explained less variation of the HEI-2005 than versions of NRF which took added sugars into account. Conversely, researchers who validated NRF models against the DHD-index found that the versions including total sugars performed slightly better than the ones including added sugars [48]. Our results are consistent with the ones from Fulgoni et al. [27], with the NRF 6.3 performing better when free sugars are included in the algorithm. This could be explained by the fact this model was originally built to include added sugars, which are a component of free sugars, as opposed to total sugars in its algorithm. Regarding the HSR specifically, 2 studies have evaluated its capacity to discriminate between core and discretionary foods when added sugars were used in the algorithm instead of total sugars [52,53]. Both studies found that using added sugars improved the performance of the HSR, with more core foods receiving ratings corresponding to a higher nutritional quality. Most will agree that adapting NP model algorithms in order to take free or added sugars into account would be more aligned with current dietary guidelines from many countries which target that nutrient. However, the mixed observations from previous studies [27,48,52,53], combined with the limited improvements in NP models performance observed in this study, suggest that it may not be worth yet to adapt NP models, particularly given that it would often require to invest time and resources in the estimation of free or added sugar content in foods. Indeed, such data are still not readily available in food databases from many jurisdictions at this time. Keep using total sugars in NP models' algorithms therefore appears to be the most appropriate approach so far, given the aforementioned scientific and practical considerations. Furthermore, the similar results observed in the current study when using total or free sugars could be explained by the strong correlation (*r* = 0.79) previously observed between the consumption of these 2 types of sugars in the studied sample [22].

This study has some strengths and limitations. First, the cross-sectional design does not allow to establish causal associations between the studied variables. This has also prevented us from evaluating the predictive validity of NP models, which is the most robust form of validity testing [7,9]. Nevertheless, multiple forms of validity testing were performed for multiple NP models simultaneously, which is a strength compared to previous studies, which often reported the results of a single type of validity testing, for a single model. Only 1 other study was identified in a recent systematic review as having simultaneously evaluated 2 types of validity for 1 of our tested models, namely the Nutri-Score [54]. The present study, by including multiple NP models, therefore allowed to better compare NP models between themselves. Second, the current data cannot be generalized to the overall population of Québec, because the sample had a higher education level, household income, and physical activity level than the average population and because missing data on covariates were not imputed. Third, because NP model validation studies often rely on previously collected data from various cohorts, it is quite difficult to end up with a precise power and sample size calculation for such studies. We therefore had no choice but to use the data available in our jurisdiction. Still, the detection of statistically significant small effect sizes suggests that the sample of over 1000 participants was sufficient. As mentioned earlier, a statistician was also consulted to confirm that the sample size was sufficient to adjust for all identified covariates. Fourth, the 24-h dietary recall instrument is prone to systematic bias, although less than other commonly used instruments such as food frequency questionnaires or screeners [[55], [56], [57]]. In addition, by using data from 2 or 3 24-h dietary recalls as opposed to only 1 recall, error was minimized. In fact, most participants completed 3 recalls, which reduces within-person variability. Fifth, the application of NP model algorithms required some nutritional data to be estimated because they were not readily available in the nutritional composition database. This was the case for the FVNL component of the HSR and Nutri-Score, as well as for the free sugar content of foods. However, to minimize risk of subjectivity, biases and errors in the estimations, the FVNL component was estimated in a double coding manner, using methods previously published in the scientific literature. Finally, other strengths of this study include the rigorous procedure through which NP models have been selected, the use of population-based data to validate the NP models, the use of biomarkers in the assessments, which represent objective data as opposed to self-report data, and the use of HEFI-2019, which is a diet quality index specifically developed for the Canadian population.

In conclusion, versions of the HSR, Nutri-Score, and NRF 6.3 NP models, which take total sugars into account, are associated with diet quality, as determined by the HEFI-2019, and with some biomarkers of health status in a French-speaking sample of the province of Québec. Including free sugars instead of total sugars in the NP model algorithms has little to no effect on the observed associations. In addition, between the 3 models tested, no model stands out as performing better than the others. Given the cross-sectional nature of this study, it would be necessary to evaluate the NP models in a prospective cohort from Québec or Canada to get a more comprehensive and global evaluation of the NP models’ validity. The results from this study will eventually be useful to the Food Quality Observatory to help identify the NP model that is best suited for characterizing and following the nutritional quality of the food supply in the Canadian context.

## Authors contributions

The authors’ contributions were as follows – M-EL, SL: designed the research; AC, MT: calculated HSR, Nutri-Score, and NRF 6.3 scores and conducted research; AB: provided free sugar data; AC: analyzed data; AC, M-EL: wrote the article; M-EL: had primary responsibility for final content; and all authors: have read and approved the final manuscript.

## Data availability

Data described in the manuscript, code book, and analytic code will be made available upon request to the corresponding author.

## Funding

Supported by the *Programme de soutien stratégique des activités de recherche de la FSAA* (to AC), FSAA Starting Funds *Soutien facultaire – Jeune Chercheur* (DC120930; to M-EL and AC); Fonds de recherche du Québec-Santé (FRQS) – COVID Supplement for *Chercheurs-boursiers* : Funding support for students (GQ131235; to M-EL and AC); Diabète Québec (FO122915; to M-EL and AC), CIHR (PJT-178070; to M-EL and SL), and FRQS – Chercheur-boursier Junior 1 Salary Award (FQ121840; to M-EL). The funders had no role in the design of the study; in the collection, analyses, or interpretation of data; in the writing of the manuscript, or in the decision to publish the results.

## Conflicts of interest

The authors declare no conflicts of interests.
