# Supplementary material for: Does the Inclusion of Free Sugars as Opposed to Total Sugars in Nutrient Profiling Models Improve Their Performance? A Cross-sectional Analysis From the PREDISE Study
Source: J Nutr. 2025 May 24;155(7):2459–74. doi: 10.1016/j.tjnut.2025.05.038 (PMC12308136; doi:10.1016/j.tjnut.2025.05.038)
Supplement: Multimedia component 1 [file mmc1.docx]

Supplementary Table 1.

Summary of each NP models’ algorithm

| **Nutrient profiling model** | **Positive components** | **Negative components** | **Final score** |
| --- | --- | --- | --- |
| Health Star Rating | Protein (g)  Fiber (g)  FVNL (%) | Energy (kcal)  Saturated fat (g)  Total sugar (g)  Sodium (mg) | Negative components – Positive components =  Baseline points – V points – P points – F points =  score between -38 to 98 points  Baseline = energy, saturated fat, total sugar, sodium  V = FVNL  P = protein  F = fiber |
| Nutri-Score | Protein (g)  Fiber (g)  FVNL (%) | Energy (kcal)  Saturated fat (g)  Total sugar (g)  Sodium (mg) | Negative components – Positive components =  Baseline points – V points – P points – F points =  score between -15 to 40 points  Baseline = energy, saturated fat, total sugar, sodium  V = FVNL  P = protein  F = fiber |
| NRF 6.3 | Protein (g)  Fiber (g)  Vitamin A (UI)  Vitamin C (mg)  Calcium (mg)  Iron (mg) | Saturated fat (g)  Total sugar^1^ (g)  Sodium (mg) | Positive components/DV – Negative components/DV = (protein/50 + fiber/25 + vitamin A/1000 + vitamin C/60 + calcium/1000 + iron/18) – (saturated fat/20 + total sugar/100 + sodium/2400) =  score between -300 and 600 points |

*DV*, daily value; *FVNL,* fruits, vegetables, nuts, and legumes.

^1^In the aim to compare the three NP models between themselves, we used total sugar instead of added sugar in the original NRF 6.3 algorithm.

Supplementary Table 2.

Sugar content thresholds for the original (including total sugars) and modified (including free sugars) versions of the Health Star Rating System and the Nutri-Score

| **Points** | **Total sugars thresholds (g/100 g)** | | | | **Free sugars thresholds (g/100 g)** | | | |
| --- | --- | --- | --- | --- | --- | --- | --- | --- |
|  | Health Star Rating^1^ | | Nutri-Score | | Health Star Rating^1^ | | Nutri-Score | |
|  | Food/Beverage  (Categories 1, 1D, 2, and 2D) | Food/Beverage  (Categories 3 and 3D) | Food | Beverage | Food/Beverage  (Categories 1, 1D, 2, and 2D) | Food/Beverage  (Categories 3 and 3D) | Food | Beverage |
| 0 | ≤5.0 | ≤5.0 | ≤4.5 | ≤0 | ≤1.9 | ≤1.9 | ≤0 | ≤0 |
| 1 | >5.0 | >5.0 | >4.5 | ≤1.5 | >1.9 | >1.9 | ≤1.5 | ≤1.5 |
| 2 | >9.0 | >9.0 | >9 | ≤3 | >3.8 | >3.8 | ≤3 | ≤3 |
| 3 | >13.5 | >13.5 | >13.5 | ≤4.5 | >5.7 | >5.7 | ≤4.5 | ≤4.5 |
| 4 | >18.0 | >18.0 | >18 | ≤6 | >7.6 | >7.6 | ≤6 | ≤6 |
| 5 | >22.5 | >22.5 | >22.5 | ≤7.5 | >9.5 | >9.5 | ≤7.5 | ≤7.5 |
| 6 | >27.0 | >27.0 | >27 | ≤9 | >11.4 | >11.4 | ≤9 | ≤9 |
| 7 | >31.0 | >31.0 | >31 | ≤10.5 | >13.3 | >13.3 | ≤10.5 | ≤10.5 |
| 8 | >36.0 | >36.0 | >36 | ≤12 | >15.2 | >15.2 | ≤12 | ≤12 |
| 9 | >40.0 | >40.0 | >40 | ≤13.5 | >17.1 | >17.1 | ≤13.5 | ≤13.5 |
| 10 | >45.0 | >45.0 | >45 | >13.5 | >19 | >19 | >13.5 | >13.5 |
| 11 | >49.0 | - | - | - | > 20.9 | - | - | - |
| 12 | >54.0 | - | - | - | >22.8 | - | - | - |
| 13 | >58.0 | - | - | - | >24.7 | - | - | - |
| 14 | >63.0 | - | - | - | >26.6 | - | - | - |
| 15 | >67.0 | - | - | - | >28.5 | - | - | - |
| 16 | >72.0 | - | - | - | >30.4 | - | - | - |
| 17 | >76.0 | - | - | - | >32.3 | - | - | - |
| 18 | >81.0 | - | - | - | >34.2 | - | - | - |
| 19 | >85.0 | - | - | - | >36.1 | - | - | - |
| 20 | >90.0 | - | - | - | >38 | - | - | - |
| 21 | >94.0 | - | - | - | >39.9 | - | - | - |
| 22 | >99.0 | - | - | - | >41.8 | - | - | - |

^1^Category 1, beverages; Category 1D, dairy beverages; Category 2, foods; Category 2D, dairy foods; Category 3, oils and fats; Category 3D, cheese.

Supplementary Table 3.

Linear regression between nutrient profiling-derived individual scores for the original and modified versions of the Health Star Rating system and HEFI-2019 as well as biomarkers of cardiometabolic risk^1^

|  | Health Star Rating original version^2^ | | | | |  | Health Star Rating modified version^2^ | | | | |
| --- | --- | --- | --- | --- | --- | --- | --- | --- | --- | --- | --- |
|  | β | SD | p | STB | Adjusted R^2^ |  | β | SD | p | STB | Adjusted R^2^ |
| HEFI-2019 | -2.02 | 0.085 | <0.0001 | -0.64 | 0.49 |  | -2.00 | 0.084 | <0.0001 | -0.64 | 0.50 |
| BMI^3^, kg/m^2^ | 0.36 | 0.061 | <0.0001 | 0.18 | 0.12 |  | 0.35 | 0.060 | <0.0001 | 0.18 | 0.12 |
| Waist circumference, cm | 0.12 | 0.061 | 0.04 | 0.02 | 0.89 |  | 0.11 | 0.060 | 0.07 | 0.02 | 0.89 |
| Body fat, % | 0.06 | 0.040 | 0.12 | 0.02 | 0.86 |  | 0.06 | 0.040 | 0.12 | 0.02 | 0.86 |
| DBP, mmHg | 0.24 | 0.100 | 0.02 | 0.07 | 0.29 |  | 0.22 | 0.099 | 0.02 | 0.07 | 0.29 |
| SBP, mmHg | 0.14 | 0.127 | 0.26 | 0.03 | 0.30 |  | 0.12 | 0.127 | 0.33 | 0.03 | 0.30 |
| Total cholesterol, mmol/L | 0.01 | 0.011 | 0.37 | 0.03 | 0.08 |  | 0.01 | 0.011 | 0.35 | 0.03 | 0.08 |
| LDL cholesterol, mmol/L | 0.004 | 0.010 | 0.68 | 0.01 | 0.04 |  | 0.004 | 0.010 | 0.65 | 0.02 | 0.04 |
| HDL cholesterol^4^, mmol/L | -0.005 | 0.003 | 0.09 | -0.05 | 0.15 |  | -0.004 | 0.003 | 0.15 | -0.04 | 0.14 |
| TG^4^, mmol/L | 0.02 | 0.005 | 0.001 | 0.10 | 0.16 |  | 0.01 | 0.005 | 0.003 | 0.09 | 0.16 |
| Fasting glucose^4^, mmol/L | 0.002 | 0.001 | 0.15 | 0.04 | 0.15 |  | 0.002 | 0.001 | 0.18 | 0.04 | 0.15 |
| Fasting insulin^4^, pmol/L | 0.01 | 0.005 | 0.01 | 0.08 | 0.22 |  | 0.01 | 0.005 | 0.03 | 0.07 | 0.22 |
| HOMA-IR^4^ | 0.01 | 0.005 | 0.01 | 0.08 | 0.25 |  | 0.01 | 0.005 | 0.02 | 0.07 | 0.25 |
| CRP^4^, mg/mL | 0.009 | 0.009 | 0.36 | 0.03 | 0.28 |  | 0.01 | 0.009 | 0.39 | 0.02 | 0.28 |
| Adiponectin^4^, ng/mL | -0.004 | 0.004 | 0.43 | -0.02 | 0.26 |  | -0.003 | 0.004 | 0.46 | -0.02 | 0.26 |

Abbreviations: *β*, beta coefficient; *BMI*, body mass index; *CRP*, C-reactive protein; *DBP*, diastolic blood pressure; *HDL*, high-density lipoproteins; *HEFI-2019*, healthy eating food index-2019; *HOMA-IR*, homeostasis model assessment of insulin resistance; *LDL*, low-density lipoproteins; *SBP*, systolic blood pressure; *SD*, standard deviation; *STB*, standardized *β* coefficient; *TG*, triglycerides. ^1^Multivariable linear models provide regression coefficients (β or STB) for outcome variables for a 1-point increase in nutrient profiling-derived scores adjusted for sex, age, administrative region, education level, ethnicity, BMI, smoking status, reporting status, and alcohol intake. The number of participants with data available for the analyses differed depending on the outcome: HEFI-2019 and BMI (*n* = 1005); waist circumference, DBP, and SBP (*n* = 1004); body fat (*n* = 1002); total cholesterol, LDL cholesterol, HDL cholesterol, TG, and fasting glucose (*n* = 995); fasting insulin and HOMA-IR (*n* = 993); CRP (*n* = 942); and adiponectin (*n* = 1001).

^2^A lower score indicates a better quality of foods consumed.

^3^Not adjusted for BMI.

^4^For HDL cholesterol, TG, glucose, insulin, HOMA-IR, CRP, and adiponectin, analyses were performed on log-transformed data.

Supplementary Table 4.

Linear regression between nutrient profiling-derived individual scores for the original and modified versions of the Nutri-Score and HEFI-2019 as well as biomarkers of cardiometabolic risk^1^

|  | Nutri-Score original version^2^ | | | | |  | Nutri-Score modified version^2^ | | | | |
| --- | --- | --- | --- | --- | --- | --- | --- | --- | --- | --- | --- |
|  | β | SD | p | STB | Adjusted R^2^ |  | β | SD | p | STB | Adjusted R^2^ |
| HEFI-2019 | -2.87 | 0.094 | <0.0001 | -0.70 | 0.55 |  | -2.56 | 0.085 | <0.0001 | -0.69 | 0.53 |
| BMI^3^, kg/m^2^ | 0.48 | 0.082 | <0.0001 | 0.19 | 0.12 |  | 0.42 | 0.074 | <0.0001 | 0.18 | 0.12 |
| Waist circumference, cm | 0.18 | 0.078 | 0.02 | 0.03 | 0.89 |  | 0.20 | 0.070 | 0.004 | 0.03 | 0.89 |
| Body fat, % | 0.08 | 0.055 | 0.13 | 0.02 | 0.86 |  | 0.09 | 0.049 | 0.06 | 0.02 | 0.86 |
| DBP, mmHg | 0.30 | 0.129 | 0.02 | 0.07 | 0.29 |  | 0.27 | 0.115 | 0.02 | 0.07 | 0.29 |
| SBP, mmHg | 0.14 | 0.168 | 0.40 | 0.02 | 0.30 |  | 0.12 | 0.155 | 0.44 | 0.02 | 0.30 |
| Total cholesterol, mmol/L | 0.01 | 0.014 | 0.37 | 0.03 | 0.08 |  | 0.01 | 0.013 | 0.41 | 0.03 | 0.08 |
| LDL cholesterol, mmol/L | 0.01 | 0.013 | 0.48 | 0.02 | 0.04 |  | 0.01 | 0.012 | 0.51 | 0.02 | 0.04 |
| HDL cholesterol^4^, mmol/L | -0.01 | 0.004 | 0.01 | -0.08 | 0.15 |  | -0.01 | 0.003 | 0.005 | -0.09 | 0.15 |
| TG^4^, mmol/L | 0.02 | 0.007 | 0.002 | 0.10 | 0.16 |  | 0.02 | 0.006 | 0.001 | 0.10 | 0.16 |
| Fasting glucose^4^, mmol/L | 0.001 | 0.002 | 0.76 | 0.01 | 0.14 |  | 0.001 | 0.002 | 0.74 | 0.01 | 0.14 |
| Fasting insulin^4^, pmol/L | 0.02 | 0.006 | 0.047 | 0.09 | 0.23 |  | 0.02 | 0.006 | 0.004 | 0.09 | 0.23 |
| HOMA-IR^4^ | 0.02 | 0.007 | 0.01 | 0.08 | 0.25 |  | 0.02 | 0.006 | 0.009 | 0.08 | 0.25 |
| CRP^4^, mg/mL | 0.02 | 0.012 | 0.11 | 0.05 | 0.29 |  | 0.02 | 0.011 | 0.04 | 0.06 | 0.29 |
| Adiponectin^4^, ng/mL | -0.01 | 0.006 | 0.35 | -0.03 | 0.26 |  | -0.005 | 0.005 | 0.32 | -0.03 | 0.26 |

Abbreviations: *β*, beta coefficient; *BMI*, body mass index; *CRP*, C-reactive protein; *DBP*, diastolic blood pressure; *HDL*, high-density lipoproteins; *HEFI-2019*, healthy eating food index-2019; *HOMA-IR*, homeostasis model assessment of insulin resistance; *LDL*, low-density lipoproteins; *SBP*, systolic blood pressure; *SD*, standard deviation; *STB*, standardized *β* coefficient; *TG*, triglycerides. ^1^Multivariable linear models provide regression coefficients (β or STB) for outcome variables for a 1-point increase in nutrient profiling-derived scores adjusted for sex, age, administrative region, education level, ethnicity, BMI, smoking status, reporting status, and alcohol intake. The number of participants with data available for the analyses differed depending on the outcome: HEFI-2019 and BMI (*n* = 1005); waist circumference, DBP, and SBP (*n* = 1004); body fat (*n* = 1002); total cholesterol, LDL cholesterol, HDL cholesterol, TG, and fasting glucose (*n* = 995); fasting insulin and HOMA-IR (*n* = 993); CRP (*n* = 942); and adiponectin (*n* = 1001).

^2^A lower score indicates a better quality of foods consumed.

^3^Not adjusted for BMI.

^4^For HDL cholesterol, TG, glucose, insulin, HOMA-IR, CRP, and adiponectin, analyses were performed on log-transformed data.

Supplementary Table 5.

Linear regression between nutrient profiling-derived individual scores for the original and modified versions of the Nutrient Rich-Food Index 6.3 and HEFI-2019 as well as biomarkers of cardiometabolic risk^1^

|  | Nutrient Rich Food 6.3 original version^2^ | | | | |  | Nutrient Rich Food 6.3 modified version^2^ | | | | |
| --- | --- | --- | --- | --- | --- | --- | --- | --- | --- | --- | --- |
|  | β | SD | p | STB | Adjusted R^2^ |  | β | SD | p | STB | Adjusted R^2^ |
| HEFI-2019 | 0.81 | 0.039 | <0.0001 | 0.59 | 0.43 |  | 0.82 | 0.031 | <0.0001 | 0.67 | 0.52 |
| BMI^3^, kg/m^2^ | -0.16 | 0.025 | <0.0001 | -0.18 | 0.12 |  | -0.14 | 0.023 | <0.0001 | -0.19 | 0.12 |
| Waist circumference, cm | -0.04 | 0.025 | 0.09 | -0.02 | 0.89 |  | -0.05 | 0.022 | 0.03 | -0.02 | 0.89 |
| Body fat, % | -0.04 | 0.019 | 0.048 | -0.03 | 0.86 |  | -0.04 | 0.017 | 0.03 | -0.03 | 0.86 |
| DBP, mmHg | -0.08 | 0.040 | 0.04 | -0.06 | 0.29 |  | -0.08 | 0.036 | 0.02 | -0.07 | 0.29 |
| SBP, mmHg | -0.04 | 0.054 | 0.50 | -0.02 | 0.30 |  | -0.04 | 0.048 | 0.42 | -0.02 | 0.30 |
| Total cholesterol, mmol/L | -0.002 | 0.005 | 0.75 | -0.01 | 0.08 |  | -0.003 | 0.004 | 0.52 | -0.02 | 0.08 |
| LDL cholesterol, mmol/L | 0.002 | 0.004 | 0.60 | 0.02 | 0.04 |  | 0.0003 | 0.004 | 0.93 | 0.003 | 0.04 |
| HDL cholesterol^4^, mmol/L | 0.002 | 0.001 | 0.19 | 0.04 | 0.14 |  | 0.002 | 0.001 | 0.08 | 0.05 | 0.15 |
| TG^4^, mmol/L | -0.01 | 0.002 | 0.002 | -0.09 | 0.16 |  | -0.01 | 0.002 | 0.001 | -0.10 | 0.16 |
| Fasting glucose^4^, mmol/L | -0.0002 | 0.0006 | 0.77 | -0.01 | 0.14 |  | 0.00001 | 0.0005 | 0.99 | 0.001 | 0.14 |
| Fasting insulin^4^, pmol/L | -0.003 | 0.002 | 0.17 | -0.04 | 0.22 |  | -0.003 | 0.002 | 0.06 | -0.05 | 0.22 |
| HOMA-IR^4^ | -0.003 | 0.002 | 0.21 | -0.04 | 0.24 |  | -0.003 | 0.002 | 0.11 | -0.05 | 0.25 |
| CRP^4^, mg/mL | -0.01 | 0.004 | 0.07 | -0.05 | 0.29 |  | -0.01 | 0.004 | 0.03 | -0.06 | 0.29 |
| Adiponectin^4^, ng/mL | 0.001 | 0.002 | 0.78 | 0.01 | 0.26 |  | 0.001 | 0.002 | 0.76 | 0.01 | 0.26 |

Abbreviations: *β*, beta coefficient; *BMI*, body mass index; *CRP*, C-reactive protein; *DBP*, diastolic blood pressure; *HDL*, high-density lipoproteins; *HEFI-2019*, healthy eating food index-2019; *HOMA-IR*, homeostasis model assessment of insulin resistance; *LDL*, low-density lipoproteins; *SBP*, systolic blood pressure; *SD*, standard deviation; *STB*, standardized *β* coefficient; *TG*, triglycerides. ^1^Multivariable linear models provide regression coefficients (β or STB) for outcome variables for a 1-point increase in nutrient profiling-derived scores adjusted for sex, age, administrative region, education level, ethnicity, BMI, smoking status, reporting status, and alcohol intake. The number of participants with data available for the analyses differed depending on the outcome: HEFI-2019 and BMI (*n* = 1005); waist circumference, DBP, and SBP (*n* = 1004); body fat (*n* = 1002); total cholesterol, LDL cholesterol, HDL cholesterol, TG, and fasting glucose (*n* = 995); fasting insulin and HOMA-IR (*n* = 993); CRP (*n* = 942); and adiponectin (*n* = 1001).

^2^A higher score indicates a better quality of foods consumed.

^3^Not adjusted for BMI.

^4^For HDL cholesterol, TG, glucose, insulin, HOMA-IR, CRP, and adiponectin, analyses were performed on log-transformed data.
